# Burns of the Eye

**Published:** 1878-10

**Authors:** 


### BURNS OF THE EYE.

To-day, two more unfortunates from the rolling mill applied at the morning clinic with terribly burned eyes, occasioned by red-hot scales flying from the rollers or trip hammer into their eyes.

The *cornea* and *sclerotic* were burned so severely as to require skillful management to prevent adhesion between the eye and the lid. Should such an attachment form, an intricate surgical operation becomes necessary to render it possible for the patient to open his eye. Then, the severe injury done the clear portion of the eye is apt to leave an opaque spot that will impair the vision or perhaps destroy it entirely. We desire to warn those who are subject to such accidents, that no time should be lost in obtaining competent advice. Do not trust to home applications in subduing the inflammation. We have seen very many eyes destroyed not only by delay in securing proper advice, but also by treatment instituted at the hands of incompetent persons.

Should the cornea be allowed to slough or ulcerate, a long and painful course of inflammation will follow, necessitating the patient to be idle for months, when a few days of skillful treatment in the beginning would restore him to his work again.

Poultices do great injury to the burned parts by increasing the sloughing process; yet this is a favorite application recommended by the patients' friends, and occasionally by a physician who should know better.

Apply cloths wrung out in ice water, avoiding all manner of poultices or hot dressings. Apply for further treatment to an occulist, as speedily as possible, who will at once take means to prevent the adhesion of the lids to the globe, or the ulceration of the *cornea*.

We desire simply to impress upon your minds the necessity of prompt action in seeking good advice and treatment where the eye is burned by scales of hot iron, or from acids coming in contact with it.

